# Optimizing Intermittent Catheterization Following Spinal Cord Injury: A Narrative Review Using the WHO Health System Building Blocks Framework

**DOI:** 10.1155/aiu/5056389

**Published:** 2026-08-03

**Authors:** Yacoub Abuzied, Aidalina Mahmud, Rasmieh Al-Amer, Azura Abdul Halain, Salimah Japar

**Affiliations:** ^1^ Department of Nursing, Faculty of Medicine and Health Sciences, Universiti Putra Malaysia, Serdang, Malaysia, upm.edu.my; ^2^ Department of Nursing, Rehabilitation Hospital, King Fahad Medical City, Riyadh, Saudi Arabia, kfmc.med.sa; ^3^ Department of Community Health, Faculty of Medicine and Health Sciences, Universiti Putra Malaysia, Serdang, Malaysia, upm.edu.my; ^4^ Faculty of Nursing, Yarmouk University, Irbid, Jordan, yu.edu.jo

**Keywords:** health service delivery, health systems strengthening, intermittent catheterization, neurogenic bladder dysfunction, spinal cord injury, WHO Health System Building Blocks

## Abstract

**Objectives:**

This narrative review examines intermittent catheterization (IC) for individuals with spinal cord injury (SCI) from a health systems perspective. Although IC is considered the preferred standard of care for neurogenic bladder after SCI, its long‐term effectiveness depends on healthcare systems’ ability to provide integrated, sustainable, and patient‐centered care. The review explores system‐level challenges and strategies to optimize IC delivery using the World Health Organization (WHO) Health System Building Blocks framework.

**Methods:**

A structured literature search was conducted across CINAHL, MEDLINE, PubMed, Scopus, ScienceDirect, and Google Scholar for studies published between January 2015 and June 2026. Relevant evidence addressing clinical outcomes, healthcare delivery, workforce capacity, access to catheter technologies, financing, and governance was synthesized using the WHO Health System Building Blocks framework. Key studies informing the review are summarized in Table 1.

**Results:**

Significant systemic barriers to effective IC implementation remain despite strong clinical evidence supporting its use. Key challenges included limited access to catheter supplies, inadequate workforce training, fragmented follow‐up services, and inconsistent patient education. Across the reviewed literature, service delivery, workforce capacity, and access to essential technologies emerged as the most influential determinants of successful IC implementation and long‐term adherence. The findings further indicated that successful IC delivery depends on the interaction and integration of multiple health system domains rather than any single component in isolation.

**Conclusion:**

Optimizing IC outcomes in SCI requires stronger health systems that support coordinated, accessible, and sustainable care. Integrating IC services into broader health system strengthening initiatives may reduce preventable complications, improve quality of life (QoL), and support Sustainable Development Goal 3 (SDG 3) on health and well‐being.

## 1. Introduction

Spinal cord injury (SCI) occurs when damage to the spinal cord leads to significant physical and functional impairments that can substantially affect daily living [1]. Bladder dysfunction is one of the most common consequences of SCI and varies according to the level and severity of injury [2]. Disruption of neural pathways involved in micturition may result in neurogenic bladder dysfunction, and in severe cases, bladder areflexia with loss of detrusor contractility [3]. Consequently, affected individuals are at increased risk of urinary tract infections (UTIs), incontinence, and upper urinary tract deterioration, including renal impairment [4].

Neurogenic bladder dysfunction is a common and clinically significant consequence of SCI, affecting more than 200,000 individuals in the United States, with approximately 10,000 new SCI cases reported annually [5]. Globally, over 500,000 new SCI cases occur each year, representing a significant public health burden [6]. Following SCI, approximately 70%–84% of individuals require catheter‐based bladder management due to loss of voluntary voiding function [6]. Effective bladder management is therefore essential to preserve renal function, prevent infections, and improve quality of life (QoL) [4].

Intermittent catheterization (IC) is widely recommended as a safe and effective method of bladder emptying, typically performed 4–6 times daily depending on the severity of bladder dysfunction [7–9]. It reduces the risk of complications such as hydronephrosis, vesicoureteral reflux, UTIs, and urinary incontinence [10]. Available catheter types include uncoated polyvinyl chloride (PVC) catheters requiring manual lubrication and hydrophilic‐coated catheters that are prelubricated and ready for use [11]. Although indwelling urinary catheters were historically the predominant approach to bladder management following SCI, IC has increasingly become the preferred method because of its superior safety profile and improved long‐term outcomes [12].

Beyond clinical technique, the effectiveness and sustainability of IC programs are strongly influenced by health system factors, including service delivery structures, workforce competency, access to essential supplies and technologies, and continuity of care.

Over the recent years, IC practice has evolved significantly within healthcare systems through multidisciplinary involvement in rehabilitation and urological care [13]. Compared to indwelling catheters, IC is associated with lower infection rates and fewer long‐term complications. It involves periodic bladder emptying through catheter insertion, followed by immediate removal, thereby avoiding permanent device dependency [14]. Additional benefits include improved patient autonomy, reduced healthcare costs, enhanced sexual health, and better control of lower urinary tract symptoms such as urgency and incontinence [10, 15]. IC also supports improved mobility, QoL, and community participation, while enhancing psychological well‐being by increasing patient involvement in self‐care and awareness of complications such as urinary retention and autonomic dysreflexia (AD). Furthermore, it promotes independence by enabling patients to manage their bladder function [16].

Despite these advantages, IC implementation in SCI care continues to face multiple physical, psychological, educational, financial, systemic, and sociocultural barriers [17]. These challenges negatively affect adherence, safety, and long‐term outcomes. Evidence indicates that approximately 50% of IC practices remain noncompliant with clinical guidelines, highlighting a persistent gap between evidence‐based recommendations and real‐world implementation [18]. Peer and professional support systems have been shown to improve coping, engagement, and self‐management [19].

Within the framework of Sustainable Development Goal 3 (SDG 3) (good health and well‐being), optimizing IC services for individuals with SCI is essential to achieving equitable access to safe, effective, and sustainable healthcare. However, there remains a limited synthesis integrating IC implementation within health system strengthening and SDG‐aligned frameworks. To our knowledge, this is the first narrative review to examine IC following SCI through the World Health Organization (WHO) Health System Building Blocks framework while explicitly linking service delivery considerations to SDG 3. By adopting a systems perspective, this review extends beyond the clinical effectiveness of IC to examine how interactions among health system domains influence the implementation, accessibility, and long‐term sustainability of care for individuals with SCI. This perspective recognizes that improvements in IC delivery depend on coordinated action across multiple health system domains, as deficiencies in one component may adversely affect the performance of others.

Accordingly, this narrative review explores system‐level strategies to improve bladder management services for individuals with SCI by strengthening access to essential supplies, enhancing patient education, improving continuity of care, optimizing service coordination, and promoting cost‐effective and sustainable IC delivery. It also evaluates how healthcare systems can provide comprehensive, integrated, and patient‐centered care for individuals living with SCI.

## 2. Materials and Methods

A comprehensive literature search was conducted across PubMed, MEDLINE, CINAHL, Scopus, ScienceDirect, and Google Scholar to identify studies published between January 2015 and June 2026. The search strategy combined terms related to SCI, IC, and health systems, including the keywords “spinal cord injury,” “intermittent catheterization,” “clean intermittent catheterization,” “health systems,” “service delivery,” “health workforce,” “health information systems,” “medical products and technologies,” “financing,” and “governance,” using Boolean operators (AND/OR). An example search strategy included combinations such as (“spinal cord injury” OR SCI) AND (“intermittent catheterization” OR “clean intermittent catheterization”) AND (“health systems” OR “service delivery” OR “health workforce” OR “health information systems” OR financing OR governance). Where applicable, proximity operators were used to refine the retrieval of relevant studies.

Titles and abstracts were initially screened for relevance, followed by full‐text assessment of potentially eligible articles. Studies were selected based on their relevance to the review objectives and their contribution to understanding the clinical, organizational, and health system dimensions of IC delivery following SCI. Study selection and data extraction were performed iteratively to identify evidence most relevant to the objectives of the review and the application of the WHO Health System Building Blocks framework.

Only peer‐reviewed English‐language articles were included to enhance methodological rigor. Studies focusing solely on the technical aspects of IC, isolated case reports, opinion pieces, editorials, and conference abstracts without full‐text availability were excluded due to insufficient methodological depth and limited relevance to the review objectives.

A total of 82 records were identified, of which 11 articles met the eligibility criteria and were included in the narrative synthesis. The included studies represented diverse evidence sources addressing clinical outcomes, patient experiences, healthcare delivery models, workforce development, access to catheter technologies, and economic considerations related to IC following SCI. Representative exemplar studies are summarized in Table 1.

**TABLE 1:** Selected exemplar studies informing intermittent catheterization delivery and outcomes following spinal cord injury (2015–2026).

Author (Year)	Country	Study design	Population	Key findings related to intermittent catheterization
Prieto et al. [10]	International	Cochrane systematic review	Individuals requiring long‐term bladder management	Intermittent catheterization remains a preferred bladder management strategy for neurogenic bladder, although evidence comparing catheter types remains limited
Shamout et al. [14]	Canada	Systematic review	Patients with a neurogenic bladder	Different IC approaches and catheter types were associated with variations in patient outcomes, satisfaction, and complication rates
Ye et al. [15]	China	Systematic review and network meta‐analysis	Intermittent catheter users	Hydrophilic catheters demonstrated advantages in comfort and reduced urethral trauma compared to standard catheters
Chen et al. [4]	Taiwan	Review	Patients with chronic spinal cord injury	Effective bladder management, including IC, is essential for preventing urinary complications and preserving renal function
Holroyd [16]	United Kingdom	Narrative review	Individuals performing self‐catheterization	IC promotes independence, self‐management, quality of life, and psychosocial well‐being
Goldstine et al. [44]	United States	Qualitative review	Adults using intermittent catheterization	Identified practical, psychosocial, and lifestyle‐related challenges that influence adherence to IC
Abuzied et al. [45]	Saudi Arabia	Phenomenological study	Arab males with SCI using IC	Highlighted lived experiences, barriers, and the importance of support systems in long‐term IC use
Welk et al. [41]	Canada	Cost‐effectiveness analysis	Patients with neurogenic lower urinary tract dysfunction	Hydrophilic catheters may be cost‐effective despite higher acquisition costs, owing to reduced complications
Couchman et al. [40]	Australia	Economic evaluation	Individuals with traumatic spinal cord injury	Long‐term use of hydrophilic catheters demonstrated favorable cost‐effectiveness compared to uncoated catheters
Bianchi et al. [51]	Italy	Scoping review	Patients and healthcare professionals	Identified patient‐, provider‐, and system‐level barriers limiting successful implementation and long‐term adherence to clean intermittent catheterization
Abuzied et al. [8]	Saudi Arabia	Qualitative study	Male patients with SCI using IC	Highlighted lived experiences, challenges, and coping strategies of patients with spinal cord injury using intermittent catheterization in Riyadh, Saudi Arabia.

As a narrative review, this study did not follow a formal systematic review protocol such as PRISMA and did not include a methodological quality appraisal of the included studies. Nevertheless, a transparent and structured search and screening process was undertaken to enhance the rigor and reproducibility of the review.

The review is guided by the WHO Health System Building Blocks framework, which comprises six interrelated components: service delivery, health workforce, Health Information Systems (HIS), access to essential medical products and technologies, financing, and leadership/governance. Collectively, these components influence health system goals, including improved health outcomes, responsiveness, social and financial risk protection, and efficiency. In the present review, the WHO Health System Building Blocks framework was used as an analytical lens to examine and organize system‐level factors influencing IC delivery among individuals with SCI, including service organization, workforce capacity, resource availability, financing mechanisms, governance structures, HIS, and access to catheter supplies [20] (Table 2).

**TABLE 2:** WHO Health System Building Blocks, IC‐related components, key challenges, and opportunities for intermittent catheterization delivery following spinal cord injury.

WHO building block	IC‐related components	Key challenges	Opportunities/strategies
Service delivery	IC clinics, rehabilitation services, follow‐up care, and telehealth	Fragmented continuity of care	Community‐based IC programs and integrated follow‐up services
Health workforce	Specialized nurses, multidisciplinary team training, and rehabilitation professionals	Shortage of trained personnel	Standardized IC education and workforce training programs
Health information systems	Electronic medical records, digital follow‐up systems, and patient education platforms	Limited digital integration and fragmented data systems	Telehealth services, integrated EMRs, and patient registries
Medical products and technologies	Catheter accessibility, hydrophilic catheters, and assistive technologies	Inconsistent catheter supply and unequal access to technologies	Centralized procurement systems and improved access to advanced catheter technologies
Health financing	Insurance coverage, reimbursement policies, and catheter supply costs	High catheter‐related costs and limited reimbursement	Subsidized catheter support programs and expanded insurance coverage
Leadership and governance	Clinical policies, practice guidelines, and ethical and regulatory standards	Weak policy implementation and lack of standardized protocols	National IC guidelines, policy reforms, and standardized clinical protocols

Table 1 summarizes the representative exemplar studies informing this narrative review, whereas Table 2 presents the key components, challenges, and opportunities associated with IC delivery across the WHO Health System Building Blocks framework.

## 3. Results

This narrative review examines the Health Service Management (HSM) aspects of IC care for individuals with SCI, focusing on how healthcare systems organize, deliver, and sustain IC programs. While the clinical effectiveness of IC is well established, its implementation and long‐term success in real‐world settings are strongly influenced by system‐level factors, including service delivery structures, workforce capacity, training quality, resource allocation, safety protocols, and supportive policy environments. The reviewed literature suggests that successful implementation of IC is determined not by any single health system component but by the interaction of multiple WHO building blocks that collectively influence care delivery and patient outcomes.

By synthesizing the available evidence, this section highlights key systemic enablers and barriers influencing IC implementation and patient outcomes across diverse healthcare settings, with particular attention to organizational and structural determinants of care delivery.

Guided by the WHO Health System Building Blocks framework, six interrelated components are examined in relation to IC: service delivery, health workforce, HIS, access to essential medicines and technologies, health financing, and leadership/governance. Although each domain is discussed separately for conceptual clarity, they operate as an interconnected system in practice and collectively determine the effectiveness and sustainability of IC delivery. The reviewed evidence suggests that IC outcomes are closely linked to both the strength of individual health system components and the interactions among them, as deficiencies in one domain may adversely influence the effectiveness of others. Collectively, these interdependent components shape the quality, accessibility, and sustainability of care for individuals with SCI.

### 3.1. Service Delivery

IC is delivered across multiple care settings, including hospital‐based programs, outpatient clinics, and home‐based self‐care [15]. Hospitals and rehabilitation centers typically provide initial assessment and training, while long‐term management is often shifted to outpatient or community‐based services. However, transitions between these care settings are frequently characterized by gaps in continuity of care, including inconsistent follow‐up, limited access to ongoing support services, and geographic disparities in service availability, which may adversely affect adherence and long‐term outcomes.

Integrated models of care that combine community nursing, telehealth consultations, and home‐based support have been shown to improve adherence to IC, enhance continuity of care, and reduce avoidable hospital admissions. However, effective implementation of these models depends on coordinated action across multiple health system domains, including adequate workforce capacity, sustainable financing, digital infrastructure, and effective governance mechanisms [21].

Nurses play a central role in IC service delivery by serving as primary educators, care coordinators, and continuity‐of‐care providers across hospital, rehabilitation, and community settings [22–24].

Adequate clinical space, accessible restrooms, and private consultation areas are essential for patient training, education, and follow‐up care. Evidence suggests that physical and environmental barriers, including inaccessible facilities, limited space, and insufficient privacy, can restrict healthcare access, compromise patient dignity, and negatively affect the quality and continuity of care provided to individuals with disabilities [25].

Collectively, these findings suggest that effective IC service delivery requires integrated, accessible, and patient‐centered models of care that ensure continuity across the rehabilitation continuum and support long‐term self‐management.

### 3.2. Health Workforce

The delivery of effective IC care depends heavily on skilled nursing staff and multidisciplinary teams. Nurses play a central role in patient education, monitoring, catheterization techniques, and early identification of complications [26]. However, variations in staff competencies and the lack of standardized training protocols may result in inconsistent patient education and support [27]. In addition, workforce shortages and high staff turnover in some healthcare settings may limit access to ongoing guidance and continuity of care and can compromise the effectiveness of service delivery, patient education, and long‐term adherence to IC programs.

Strengthening workforce capacity through standardized training curricula, continuous professional development, and structured mentorship programs may improve the consistency and quality of IC care. Effective workforce development, however, also requires supportive leadership, adequate financing, and access to educational resources and technologies to ensure sustainable implementation and improved patient outcomes [28, 29].

Collectively, these findings suggest that investment in workforce development is fundamental to the successful implementation and sustainability of IC services across the continuum of SCI care.

### 3.3. HIS

HIS are essential for supporting the delivery and long‐term management of IC among individuals with SCI. Robust HIS facilitate routine data collection, documentation of catheterization practices, monitoring of complications such as UTIs, and longitudinal evaluation of patient outcomes, thereby supporting proactive and coordinated IC management [30]. Electronic Medical Record (EMR) and patient registries can improve continuity of care by enabling healthcare providers to track adherence, identify high‐risk individuals, and coordinate multidisciplinary interventions [31, 32].

Digital technologies, including mobile health applications and telehealth platforms, can further support patient education, self‐monitoring, and timely communication between patients and healthcare professionals [33]. In addition, the systematic collection and analysis of clinical data can inform quality improvement initiatives, guide resource allocation, and support evidence‐based decision‐making in IC services. The effectiveness of these information systems, however, depends on adequate workforce competencies, investment in digital infrastructure, and supportive governance and financing mechanisms. Strengthening HIS may therefore reduce information gaps, enhance care coordination, and improve long‐term outcomes for individuals using IC following SCI [34].

Collectively, these findings suggest that HIS are fundamental to monitoring outcomes, supporting coordinated care, and enabling continuous quality improvement in IC services for individuals with SCI.

### 3.4. Medical Products/Technologies

Ensuring consistent access to IC supplies is a critical component of effective neurogenic bladder management in individuals with SCI. Although IC devices are classified as medical consumables rather than medications, they fall within the broader WHO framework of essential medicines and health products [35]. Barriers to the availability, affordability, and procurement of catheters, particularly hydrophilic types, may negatively affect adherence, increase the risk of complications, and contribute to poorer clinical outcomes by limiting patients’ ability to perform IC consistently and safely [14].

Addressing these challenges through supportive policies, standardized procurement and supply systems, and equitable access strategies is essential for improving continuity of care and optimizing long‐term patient outcomes. Effective access to catheter technologies, however, also depends on adequate financing mechanisms, efficient supply chain management, workforce training, and governance structures that ensure the appropriate prescription, distribution, and monitoring of IC supplies [36].

Collectively, these findings suggest that ensuring reliable access to appropriate catheter technologies is fundamental to sustaining adherence, reducing preventable complications, and supporting patient‐centered IC care following SCI.

### 3.5. Health Financing

IC programs require careful resource planning, particularly regarding catheter supplies and associated equipment. Costs vary substantially depending on catheter type, frequency of use, and insurance or reimbursement coverage. In some healthcare systems, inconsistent funding policies and limited reimbursement mechanisms create barriers to access, particularly for individuals in rural or low‐resource settings, thereby increasing the risk of interrupted IC use and inequities in health outcomes [37].

The effectiveness of these financing strategies, however, depends on coordinated governance, efficient procurement and supply systems, and workforce capacity to ensure appropriate prescribing, distribution, and patient support [38]. Incorporating IC‐related expenses into long‐term disability funding and rehabilitation financing schemes may further enhance financial sustainability and continuity of care [39, 40].

Hydrophilic catheters are generally more expensive than standard uncoated catheters due to their prelubricated design, which reduces friction during insertion [40]. Reported costs average US $3.77 per hydrophilic catheter, compared to US $1.07 per standard uncoated catheter. Given that individuals commonly perform IC 4–6 times daily, these cost differences may create a substantial long‐term financial burden for both patients and healthcare systems, potentially influencing catheter selection, adherence, and equitable access to care, particularly when reimbursement mechanisms are inadequate or absent [41].

Collectively, these findings suggest that sustainable financing mechanisms are fundamental to ensuring equitable access to IC technologies and reducing the long‐term clinical and economic burden associated with SCI‐related bladder dysfunction.

### 3.6. Leadership/Governance

Effective IC programs depend on clear governance structures, standardized policies, and consistent implementation frameworks [42]. Although national and regional guidelines provide important direction for clinical practice, gaps frequently remain in their implementation, monitoring, and accountability at the service level, resulting in variations in service delivery and patient experiences across healthcare settings.

Policymakers and healthcare organizations should ensure that IC protocols are integrated into broader rehabilitation and chronic care strategies, supported by clear service standards and accountability mechanisms to promote safe, equitable, and patient‐centered care. Effective governance, however, also requires coordinated financing, workforce development, information systems, and access to essential technologies to ensure that policies are translated into sustainable improvements in practice [43].

Collectively, these findings suggest that strong leadership and governance are fundamental to aligning policies, resources, and service delivery processes to achieve high‐quality and sustainable IC care for individuals with SCI.

## 4. Discussion

### 4.1. Identified Challenges in Health Service Delivery to IC

Delivering IC services to individuals with SCI is often challenged by multiple systemic and contextual barriers [44, 45]. Limited availability of trained healthcare professionals, particularly nurses and rehabilitation specialists, can restrict patient education, follow‐up, and monitoring, thereby reducing adherence to IC protocols and increasing the risk of complications such as UTIs [46]. These workforce limitations are frequently compounded by inadequate service coordination, insufficient training resources, and disparities in access to specialized rehabilitation services. Access to specialized continence care is also often uneven, particularly in rural and underserved areas, contributing to disparities in service quality.

Cultural and personal factors may further affect acceptance and consistent use of IC. In some settings, urinary care and self‐catheterization may be associated with stigma or embarrassment, reducing patient engagement in care [45]. Language barriers, low health literacy, and differing beliefs regarding disability and bodily autonomy may also influence adherence to IC regimens [44]. In addition, limited access to affordable catheter supplies, poorly coordinated transitions between hospital and community care, and inadequate insurance coverage can contribute to suboptimal outcomes [47, 48]. These challenges rarely occur in isolation and often reflect interactions among deficiencies in financing, service delivery, workforce capacity, governance, and access to essential technologies, collectively undermining the sustainability and effectiveness of IC services.

Addressing these barriers therefore requires integrated health system strengthening strategies that simultaneously target structural, financial, educational, and sociocultural determinants of IC care rather than isolated interventions focused on individual components of the health system [49].

### 4.2. Opportunities for Improving Health Service Delivery to IC

Despite these challenges, several opportunities exist to strengthen IC service delivery for individuals with SCI. Multidisciplinary care models involving nurses, urologists, rehabilitation specialists, and social workers can improve continuity of care and address both clinical and psychosocial needs [50]. Expanding healthcare provider training, particularly in community and primary care settings, may enhance IC education, follow‐up, and long‐term adherence while strengthening the integration of services across the continuum of care [26, 51]. In addition, telehealth and digital health tools can improve access to follow‐up care and patient education, especially for individuals in rural or underserved areas [52].

Policy reforms and sustainable funding mechanisms are also essential to ensure equitable access to catheter supplies and related services [53]. Culturally sensitive education programs tailored to patients’ language and social context may further improve engagement and reduce stigma associated with IC use [54].

Among the six WHO building blocks, service delivery, workforce capacity, and access to essential technologies emerged as the most influential determinants of successful IC implementation. Deficiencies in these domains were consistently associated with reduced adherence, increased complications, and poorer patient experiences. Consequently, health system strengthening efforts should prioritize integrated care pathways, specialized workforce training, and sustainable catheter procurement systems. Collectively, the reviewed evidence demonstrates that although IC is clinically effective for bladder management following SCI, its long‐term success depends largely on the strength and integration of the healthcare systems supporting its delivery. Persistent barriers, including fragmented care pathways, workforce limitations, inconsistent access to supplies, and variability in guideline adherence, continue to affect care quality and patient outcomes [55, 56]. Effective IC programs require coordinated multidisciplinary services, standardized training, reliable supply systems, and sustainable financing mechanisms [57].

Importantly, the six WHO Health System Building Blocks should not be viewed as independent domains. Effective service delivery depends on a skilled workforce, reliable access to essential technologies, and robust HIS that support continuity of care and monitoring of outcomes. Similarly, financing and governance influence resource allocation, implementation of clinical protocols, and the sustainability of IC services. These interdependencies suggest that strengthening a single domain in isolation is unlikely to optimize long‐term IC outcomes following SCI, highlighting the need for integrated and coordinated health system strengthening strategies. Similarly, weak HIS may impede monitoring, quality improvement initiatives, and coordination of multidisciplinary services, thereby adversely affecting service delivery and long‐term adherence to IC.

These international experiences suggest that sustained policy commitment and coordinated investment across multiple health system domains can substantially improve the accessibility and continuity of IC services. Countries such as Sweden, Canada, Saudi Arabia, the United Kingdom, and South Korea have implemented funding models, rehabilitation programs, telehealth services, and supply support systems that enhance accessibility and patient support [45, 58–61].

National guidelines should be translated into actionable protocols at the local level, with attention to legal and ethical issues, including privacy, consent, and patient dignity [62]. Investment in digital health tools, such as telehealth consultations, electronic health records, and mobile education platforms, can expand the reach of IC services and address barriers to access due to geography and mobility [63]. In this context, tailoring services to individual patient needs and preferences is not only clinically beneficial but also a health system imperative [64]. Together, these findings underscore the need for integrated, patient‐centered, and system‐driven strategies to optimize the impact of sustainable bladder management within health systems supporting SDG 3.

Ultimately, strengthening health systems for IC delivery represents a scalable, patient‐centered, and cost‐effective strategy to improve long‐term outcomes, reduce preventable complications, and advance equitable and sustainable care for individuals living with SCI globally [40, 65].

### 4.3. Strengths and Limitations

This narrative review has several limitations. First, although a structured search strategy was used, the review did not follow a formal systematic review methodology, such as PRISMA, and no formal quality assessment of the included studies was conducted. Nevertheless, a transparent and structured search and screening process was undertaken to enhance the rigor and reproducibility of the review.

Second, only English‐language studies published in peer‐reviewed sources were included. As a result, relevant evidence published in other languages or within the gray literature may have been overlooked. In addition, the narrative review approach does not allow for quantitative synthesis of findings or estimation of effect sizes. The findings should therefore be interpreted as a broad synthesis of the available evidence rather than definitive conclusions regarding the effectiveness of specific interventions. Finally, as a narrative review, this study primarily provides an interpretive synthesis of the available literature, which limits the ability to establish causal relationships, directly compare healthcare delivery models, or determine the relative effectiveness of specific health system interventions.

Despite these limitations, this review offers several strengths. By examining IC from both clinical and health system perspectives, it provides a comprehensive overview of the factors influencing long‐term bladder management following SCI. The inclusion of qualitative, quantitative, and policy‐related literature also allowed consideration of perspectives from patients, healthcare professionals, and healthcare systems. To our knowledge, this is among the first reviews to examine IC following SCI through a health systems lens while explicitly considering the interactions among WHO health system domains and their implications for sustainable and equitable care.

Overall, the review provides practical and policy‐relevant insights that may support clinicians, healthcare leaders, and policymakers in developing more coordinated, accessible, patient‐centered, and sustainable services for individuals living with SCI.

## 5. Conclusion

This narrative review highlights that effective IC care for individuals with SCI requires the integration of both clinical practice and health system management approaches. Although IC is effective in reducing complications and preserving renal function, successful long‐term outcomes depend on appropriate patient education, coordinated multidisciplinary care, and sustained follow‐up. The findings also align IC care with SDG 3, emphasizing the importance of equitable, high‐quality, and patient‐centered healthcare services.

Persistent challenges, including workforce shortages, unequal access to catheter supplies, cultural barriers, and fragmented care pathways, continue to affect the quality and accessibility of IC services. Improving outcomes requires strengthened community‐based services, standardized training, ongoing support mechanisms, and greater integration of digital health technologies and multidisciplinary care models. Future research should evaluate healthcare delivery models, implementation strategies, and patient experiences to support scalable, evidence‐based approaches for sustainable and equitable IC care worldwide.

Therefore, interventions aimed at improving IC outcomes should adopt a systems‐based approach that simultaneously addresses workforce development, service integration, access to technologies, HIS, financing, and governance. As demonstrated throughout this review, these health system domains are interdependent, and sustainable improvements in IC delivery are more likely to be achieved through coordinated and integrated health system strengthening strategies rather than isolated interventions targeting individual components. Such an approach has the potential to improve long‐term outcomes, reduce preventable complications, and promote equitable and sustainable bladder management for individuals living with SCI worldwide.

## Author Contributions

Yacoub Abuzied initiated and planned the study and drafted the study protocol with input from all other authors. Yacoub Abuzied, Aidalina Mahmud, Rasmieh Al‐Amer, Azura Abdul Halain and Salimah Japar performed analyses. Yacoub Abuzied, Aidalina Mahmud, Rasmieh Al‐Amer, and Salimah Japar drafted the first version of the manuscript after all authors had explored the final study report.

## Funding

This research received no specific grant from any funding agency in the public, commercial, or not‐for‐profit sectors.

## Disclosure

All authors approved the final version of the manuscript for submission.

## Ethics Statement

Ethical approval was not applicable for this narrative review.

## Conflicts of Interest

The authors declare no conflicts of interest.

## Data Availability

Data sharing is not applicable to this article as no datasets were generated or analyzed during the current study.

## References

[1] Sezer N. , Akkuş S. , and Uğurlu F. G. , Chronic Complications of Spinal Cord Injury, World Journal of Orthopedics. (2015) 6, no. 1. 10.5312/wjo.v6.i1.24 PMC4303787 25621208

[2] Hamid R. , Averbeck M. A. , Chiang H. et al., Epidemiology and Pathophysiology of Neurogenic Bladder After Spinal Cord Injury, World Journal of Urology. (2018) 36, no. 10, 1517–1527, 10.1007/s00345-018-2301-z. 29752515

[3] Dellon A. L. and Herati A. S. , Review of Bladder Pain and Referred T12–L2 Input as One Etiology for Interstitial Cystitis, Journal of Reconstructive Microsurgery Open. (2019) 4, no. 02, e58–e63, 10.1055/s-0039-1696954.

[4] Chen Y-C. , Ou Y-C. , Hu J-C. et al., Bladder Management Strategies for Urological Complications in Patients With Chronic Spinal Cord Injury, Journal of Clinical Medicine. (2022) 11, no. 22, 10.3390/jcm11226850. PMC9697498 36431327

[5] Bing J. , You H. , Dai Y. , and Ding Y. , Progress and Research Trends in Neurogenic Bladder After Spinal Cord Injury Bibliometric Analysis Based on Web of Science Database: An Observational Study, Medicine. (2024) 103, no. 24, 10.1097/md.0000000000038491. PMC11175955 38875432

[6] Zlatev D. , Shem K. , and Elliott C. , How Many Spinal Cord Injury Patients Can Catheterize Their Own Bladder? the Epidemiology of Upper Extremity Function as it Affects Bladder Management, Spinal Cord. (2016) 54, no. 4, 287–291, 10.1038/sc.2015.169. 26782841

[7] Prieto J. , Murphy C. L. , Moore K. N. , and Fader M. , Intermittent Catheterisation for Long‐Term Bladder Management, Cochrane Database of Systematic Reviews. (2014) 9. 10.1002/14651858.CD006008.pub3 25208303

[8] Abuzied Y. , Al-Amer R. , Abdul Halain A. , and Japar S. , Lived Experiences, Challenges and Coping Strategies of Patients With Spinal Cord Injury Using Intermittent Catheterisation in Riyadh, Saudi Arabia: A Qualitative Study, British Medical Journal Open. (2026) 16, no. 6, 10.1136/bmjopen-2025-115907. PMC13264942 42276795

[9] Gustafson J. and Petersen T. , Bladder and Bowel Health, Essential Clinical Skills in Nursing, Johns Hopkins Medicine.

[10] Prieto J. , Murphy C. L. , Moore K. N. , and Fader M. , Intermittent Catheterisation for Long‐Term Bladder Management, Cochrane Database of Systematic Reviews. (2017) 8, 10.1002/14651858.cd006008.pub4. PMC6483323 28796279

[11] Stoffel J. T. and Yu L. , Urinary Catheters, Updates in Neurourology, An Issue of Urologic Clinics, E-Book. (2024) 51, no. 2, 253–262, 10.1016/j.ucl.2024.01.003. 38609197

[12] Feneley R. C. , Hopley I. B. , and Wells P. N. , Urinary Catheters: History, Current Status, Adverse Events and Research Agenda, Journal of Medical Engineering & Technology. (2015) 39, no. 8, 459–470, 10.3109/03091902.2015.1085600. 26383168 PMC4673556

[13] Liberati E. G. , Gorli M. , and Scaratti G. , Invisible Walls Within Multidisciplinary Teams: Disciplinary Boundaries and Their Effects on Integrated Care, Social Science & Medicine. (2016) 150, 31–39, 10.1016/j.socscimed.2015.12.002. 26730879

[14] Shamout S. , Biardeau X. , Corcos J. , and Campeau L. , Outcome Comparison of Different Approaches to Self-Intermittent Catheterization in Neurogenic Patients: A Systematic Review, Spinal Cord. (2017) 55, no. 7, 629–643, 10.1038/sc.2016.192. 28117329

[15] Ye D. , Chen Y. , Jian Z. et al., Catheters for Intermittent Catheterization: A Systematic Review and Network Meta-Analysis, Spinal Cord. (2021) 59, no. 6, 587–595, 10.1038/s41393-021-00620-w. 33911191

[16] Holroyd S. , How Intermittent Self-Catheterisation Can Promote Independence, Quality of Life and Wellbeing, British Journal of Nursing. (2018) 27, no. Sup15, S4–S10, 10.12968/bjon.2018.27.sup15.s4. 30088975

[17] Jang W-H. , Lee S-B. , Kim D-W. et al., ICT-Based Health Care Services for Individuals With Spinal Cord Injuries: A Feasibility Study, Sensors. (2020) 20, no. 9, 10.3390/s20092491. PMC7249337 32354052

[18] Yao L. , Ahmed M. M. , Guyatt G. H. et al., Discordant and Inappropriate Discordant Recommendations in Consensus and Evidence Based Guidelines: Empirical Analysis, British Medical Journal. (2021) 375, 10.1136/bmj-2021-066045. PMC8613613 34824101

[19] Dineen-Griffin S. , Garcia-Cardenas V. , Williams K. , and Benrimoj S. I. , Helping Patients Help Themselves: A Systematic Review of Self-Management Support Strategies in Primary Health Care Practice, Public Library of Science ONE. (2019) 14, no. 8, 10.1371/journal.pone.0220116. PMC6675068 31369582

[20] Organization W. H. , Monitoring the Building Blocks of Health Systems: A Handbook of Indicators and Their Measurement Strategies, 2010, WHO Document Production Services.

[21] Nasir J. A. , Hussain S. , and Dang C. , An Integrated Planning Approach Towards Home Health Care, Telehealth and Patients Group Based Care, Journal of Network and Computer Applications. (2018) 117, 30–41, 10.1016/j.jnca.2018.05.009.

[22] Nye A. I. , Experience of Nurses in Caring for Patients With Short Term Indwelling Urinary Catheters During Hospitalization: A Challenge to Reshape Caring Practices, 2018, Washington State University.

[23] Newman D. K. , Carillo M. , Talley K. , Starr J. A. , Thompson D. , and Wyman J. F. , Advancing the Roles of the Registered Nurse and Advanced Practice Registered Nurse in Continence and Pelvic Health Care in the United States: A White Paper, Urologic Nursing. (2024) 44, no. 2, 10.7257/2168-4626.2024.44.2.64.

[24] Abuzied Y. , Al-Amer R. , Abuzaid M. , and Somduth S. , The Magnet Recognition Program and Quality Improvement in Nursing, Global Journal on Quality and Safety in Healthcare. (2022) 5, no. 4, 106–108, 10.36401/jqsh-22-12. 37260930 PMC10229039

[25] Mitchell R. J. , Ryder T. , Matar K. , Lystad R. P. , Clay‐Williams R. , and Braithwaite J. , An Overview of Systematic Reviews to Determine the Impact of Socio‐Environmental Factors on Health Outcomes of People With Disabilities, Health and Social Care in the Community. (2022) 30, no. 4, 1254–1274, 10.1111/hsc.13665. 34850472

[26] Alex J. , Maneze D. , Ramjan L. M. , Ferguson C. , Montayre J. , and Salamonson Y. , Effectiveness of Nurse-Targeted Education Interventions on Clinical Outcomes for Patients With Indwelling Urinary Catheters: A Systematic Review, Nurse Education Today. (2022) 112, 10.1016/j.nedt.2022.105319. 35298974

[27] Sarre S. , Maben J. , Aldus C. et al., The Challenges of Training, Support and Assessment of Healthcare Support Workers: A Qualitative Study of Experiences in Three English Acute Hospitals, International Journal of Nursing Studies. (2018) 79, 145–153, 10.1016/j.ijnurstu.2017.11.010. 29272810

[28] Creta A. M. and Gross A. H. , Components of an Effective Professional Development Strategy: The Professional Practice Model, Peer Feedback, Mentorship, Sponsorship, and Succession Planning, Seminars in Oncology Nursing. (2020) Elsevier. 10.1016/j.soncn.2020.151024 32402725

[29] Abuzied Y. , Maymani H. , AlMatouq B. , and AlDosary O. , Reducing the Length of Stay by Enhancing the Patient Discharge Process: Using Quality Improvement Tools to Optimize Hospital Efficiency, Global Journal on Quality and Safety in Healthcare. (2021) 4, no. 1, 44–49, 10.36401/jqsh-20-27. 37260536 PMC10229011

[30] Wilde M. H. , McMahon J. M. , Fairbanks E. et al., Feasibility of a Web-Based Self-Management Intervention for Intermittent Urinary Catheter Users With Spinal Cord Injury, Journal of Wound, Ostomy and Continence Nursing. (2016) 43, no. 5, 529–538, 10.1097/won.0000000000000256. PMC5016217 27488740

[31] Bayliss E. A. , Ellis J. L. , Shoup J. A. , Zeng C. , McQuillan D. B. , and Steiner J. F. , Effect of Continuity of Care on Hospital Utilization for Seniors With Multiple Medical Conditions in an Integrated Health Care System, Annals of Family Medicine. (2015) 13, no. 2, 123–129, 10.1370/afm.1739. 25755033 PMC4369605

[32] Kruse C. S. , Stein A. , Thomas H. , and Kaur H. , The Use of Electronic Health Records to Support Population Health: A Systematic Review of the Literature, Journal of Medical Systems. (2018) 42, no. 11, 10.1007/s10916-018-1075-6. PMC6182727 30269237

[33] Kim S. , Lee K.-H. , Hwang H. , and Yoo S. , Analysis of the Factors Influencing Healthcare Professionals’ Adoption of Mobile Electronic Medical Record (EMR) Using the Unified Theory of Acceptance and Use of Technology (UTAUT) in a Tertiary Hospital, BMC Medical Informatics and Decision Making. (2015) 16, no. 1, 10.1186/s12911-016-0249-8. PMC4736616 26831123

[34] Colombo F. , Oderkirk J. , and Slawomirski L. , Health Information Systems, Electronic Medical Records, and Big Data in Global Healthcare: Progress and Challenges in OECD Countries, 2020, Handbook of Global Health, 1–31.

[35] Organization W. H. , WHO Global Model Regulatory Framework for Medical Devices Including in Vitro Diagnostic Medical Devices, 2017, WHO.

[36] Sinha K. K. and Kohnke E. J. , Health Care Supply Chain Design: Toward Linking the Development and Delivery of Care Globally, Decision Sciences. (2009) 40, no. 2, 197–212, 10.1111/j.1540-5915.2009.00229.x.

[37] Beatty K. , Heffernan M. , Hale N. , and Meit M. , Funding and Service Delivery in Rural and Urban Local US Health Departments in 2010 and 2016, American Journal of Public Health. (2020) 110, no. 9, 1293–1299, 10.2105/ajph.2020.305757. 32673110 PMC7427251

[38] Pastakia S. D. , Tran D. N. , Manji I. , Wells C. , Kinderknecht K. , and Ferris R. , Building Reliable Supply Chains for Noncommunicable Disease Commodities: Lessons Learned From HIV and Evidence Needs, Acquired Immunodeficiency Syndrome. (2018) 32, no. S1, S55–S61, 10.1097/qad.0000000000001878. 29952791

[39] Abedi A. , Sayegh A. S. , Ha N. T. et al., Health Care Economic Burden of Treatment and Rehabilitation for Neurogenic Lower Urinary Tract Dysfunction: A Systematic Review, Journal of Urology. (2022) 208, no. 4, 773–783, 10.1097/ju.0000000000002862. 35901183

[40] Couchman M. A. , Nunn A. , Delaney D. et al., Cost-Effectiveness of Long-Term Intermittent Catheterisation With Hydrophilic and Uncoated Catheters in Traumatic Spinal Cord Injury in Australia, Continence. (2022) 4, 10.1016/j.cont.2022.100513.

[41] Welk B. , Isaranuwatchai W. , Krassioukov A. , Husted Torp L. , and Elterman D. , Cost-Effectiveness of Hydrophilic-Coated Intermittent Catheters Compared With Uncoated Catheters in Canada: A Public Payer Perspective, Journal of Medical Economics. (2018) 21, no. 7, 639–648, 10.1080/13696998.2018.1443112. 29458282

[42] Schladen M. M. , Rounds A. K. , McManus T. , Bennewith A. , Claypool H. , and Groah S. L. , Intermittent Catheter Reimbursement in the United States: The Experience of Nine Stakeholders Through the Lens of Actor-Network Theory, Qualitative Report. (2021) 26, 443–464.

[43] Sabariego C. , Bickenbach J. , and Stucki G. , Supporting Evidence-Informed Policy Making in Rehabilitation: A Logic Framework for Continuous Improvement of Rehabilitation Programs, Health Policy. (2022) 126, no. 3, 152–157, 10.1016/j.healthpol.2021.11.007. 34872725

[44] Goldstine J. , Leece R. , Samas S. , and Zonderland R. , In Their Own Words: Adults’ Lived Experiences With Intermittent Catheterization, Journal of Wound, Ostomy and Continence Nursing. (2019) 46, no. 6, 513–518, 10.1097/WON.0000000000000591. 31651798

[45] Abuzied Y. , Al‐Amer R. , Saleh M. Y. , Somduth S. , AlBashtawy M. , and Ali A. M. , Exploring the Lived Experience of Arab Male Patients on Intermittent Catheterization After Spinal Cord Injury: A Phenomenological Study, International Journal of Nursing Practice. (2024) 30, no. 5, 10.1111/ijn.13268. 38798100

[46] Feldacker C. , Murenje V. , Holeman I. et al., Reducing Provider Workload While Preserving Patient Safety: A Randomized Control Trial Using 2-Way Texting for Postoperative Follow-Up in Zimbabwe’s Voluntary Medical Male Circumcision Program, JAIDS Journal of Acquired Immune Deficiency Syndromes. (2020) 83, no. 1, 16–23, 10.1097/qai.0000000000002198. 31809358 PMC6903365

[47] Thomsen P. B. , Delays in Care due to Inaccessibility of Essential Medical Items in Surgical Unit, 2024, University of San Francisco.

[48] Al-Worafi Y. M. , Healthcare Facilities in Developing Countries: Infrastructure. Handbook of Medical and Health Sciences in Developing Countries: Education, Practice, and Research, 2023, Springer, 1–21.

[49] Organization W. H. , WHO Global Strategy on People-Centred and Integrated Health Services: Interim Report, 2015, World Health Organization.

[50] Collet R. , van Grootel J. , van Dongen J. et al., The Impact of Multidisciplinary Transitional Care Interventions for Complex Care Needs: A Systematic Review and Meta-Analysis, Gerontologist. (2025) 65, no. 6, 10.1093/geront/gnaf088. PMC12086065 40040533

[51] Bianchi D. , Rosato E. , Terzoni S. , Turbanti A. , Pletto S. , and Agrò E. F. , Possible Barriers for Patients and Professionals That Prevent the Use of Clean Intermittent Catheterization: A Scoping Review, Neurourology and Urodynamics. (2026) 45, no. 1, 200–208, 10.1002/nau.70166. 41087830

[52] McLendon S. F. , Interactive Video Telehealth Models to Improve Access to Diabetes Specialty Care and Education in the Rural Setting: A Systematic Review, Diabetes Spectrum. (2017) 30, no. 2, 124–136, 10.2337/ds16-0004. 28588379 PMC5439356

[53] Al-Worafi Y. M. , Access to the Medical Devices in Developing Countries, Handbook of Medical and Health Sciences in Developing Countries: Education, Practice, and Research. (2023) Springer, 1–31.

[54] Henderson S. , Kendall E. , and See L. , The Effectiveness of Culturally Appropriate Interventions to Manage or Prevent Chronic Disease in Culturally and Linguistically Diverse Communities: A Systematic Literature Review, Health and Social Care in the Community. (2011) 19, no. 3, 225–249, 10.1111/j.1365-2524.2010.00972.x. 21208326

[55] Evans-Lacko S. , Jarrett M. , McCrone P. , and Thornicroft G. , Facilitators and Barriers to Implementing Clinical Care Pathways, BMC Health Services Research. (2010) 10, no. 1, 10.1186/1472-6963-10-182. PMC2912894 20584273

[56] Nao S. , Medicine D. M. , BoG H. , and CoItQoHC G. , Crossing the Global Quality Chasm: Improving Health Care Worldwide, 2018, National Academies Press. 30605296

[57] Organization W. H. , Continuity and Coordination of Care: A Practice Brief to Support Implementation of the WHO Framework on Integrated People-Centred Health Services, 2018, WHO.

[58] Dhakal R. , Baniya M. , Solomon R. M. et al., Telerehabilitation Nepal (TERN) for People With Spinal Cord Injury and Acquired Brain Injury: A Feasibility Study, Rehabilitation Process and Outcome. (2022) 11, 10.1177/11795727221126070. PMC9583203 36278119

[59] Kim Y. , Cho M. H. , Do K. et al., Incidence and Risk Factors of Urinary Tract Infections in Hospitalised Patients With Spinal Cord Injury, Journal of Clinical Nursing. (2021) 30, no. 13-14, 2068–2078, 10.1111/jocn.15763. 33829566

[60] Duff J. , Grant L. C. , Gilchrist H. , and Jones K. , Building and Sustaining Inpatient-Clinician Collaboration in Spinal Cord Injury Rehabilitation: A Case Example Using the Stoke Mandeville Spinal Needs Assessment Checklist (SMS-NAC) and Goal Planning Programme, Journal of Clinical Medicine. (2022) 11, no. 13, 10.3390/jcm11133730. PMC9267847 35807024

[61] Lindqvist T. , Health Economic Analysis of a Urinary Catheter Which Reduce the Frequency of Urinary Tract Infections (Utis) Research Questions: A) What is the Willingness-to-Pay (WTP) in Sweden-From a Patient Perspective-for a Urinary Catheter Which Reduce the Annual Frequency of Urinary Tract Infections by 25% and 50%, Respectively, and (B) is the WTP Dependent on the Annual Frequency of Utis and Gender?, Bachelor thesis in Public Administration. (2019) 15.

[62] Nacu A-G. , Constantin D-A. , and Rogozea L. M. , Ethical Dilemmas and Legal Responsibilities in Patient Care: An Analysis of Hospital Safety, Healthcare. (2025) MDPI. 10.3390/healthcare13212800 PMC12607453 41228168

[63] Egemba M. , Aderibigbe-Saba C. , Ajayi Simeon A. , Patrick A. , and Olufunke O. , Telemedicine and Digital Health in Developing Economies: Accessibility Equity Frameworks for Improved Healthcare Delivery, International Journal of Multidisciplinary Research and Growth Evaluation. (2020) 1, no. 5, 220–238, 10.54660/.ijmrge.2020.1.5.220-238.

[64] Zwijnenberg N. C. , Hendriks M. , Bloemendal E. et al., Patients’ Need for Tailored Comparative Health Care Information: A Qualitative Study on Choosing a Hospital, Journal of Medical Internet Research. (2016) 18, no. 11, 10.2196/jmir.4436. PMC5153531 27895006

[65] Shamout S. , Nazha S. , Dragomir A. , Baverstock R. , Corcos J. , and Campeau L. , A Cost-Effectiveness Analysis of Bladder Management Strategies in Neurogenic Lower Urinary Tract Dysfunction After Spinal Cord Injury: A Publicly Funded Health Care Perspective, Spinal Cord. (2023) 61, no. 4, 269–275, 10.1038/s41393-023-00883-5. 36894764

